# Battery-Powered RSU Running Time Monitoring and Prediction Using ML Model Based on Received Signal Strength and Data Transmission Frequency in V2I Applications

**DOI:** 10.3390/s23073536

**Published:** 2023-03-28

**Authors:** Vienna N. Katambire, Richard Musabe, Alfred Uwitonze, Didacienne Mukanyiligira

**Affiliations:** 1 African Center of Excellence in Internet of Things (ACEIoT), College of Science and Technology, University of Rwanda, Kigali P.O. Box 3900, Rwanda; 2 Rwanda Polytechnic, Kigali P.O. Box 164, Rwanda; 3 National Council for Science and Technology, Kigali P.O. Box 2285, Rwanda

**Keywords:** current consumption, roadside units, machine learning, V2I communication

## Abstract

The application of the Internet of Things (IoT), vehicles to infrastructure (V2I) communication and intelligent roadside units (RSU) are promising paradigms to improve road traffic safety. However, for the RSUs to communicate with the vehicles and transmit the data to the remote location, RSUs require enough power and good network quality. Recent advances in technology have improved lithium-ion battery capabilities. However, other complementary methodologies including battery management systems (BMS) have to be developed to provide an early warning sign of the battery’s state of health. In this paper, we have evaluated the impact of the received signal strength indication (RSSI) and the current consumption at different transmission frequencies on a static battery-based RSU that depends on the global system for mobile communications (GSM)/general packet radio services (GPRS). Machine learning (ML) models, for instance, Random Forest (RF) and Support Vector Machine (SVM), were employed and tested on the collected data and later compared using the coefficient of determination ($R^{2}$). The models were used to predict the battery current consumption based on the RSSI of the location where the RSUs were imposed and the frequency at which the RSU transmits the data to the remote database. The RF was preferable to SVM for predicting current consumption with an $R^{2}$ of 98% and 94%, respectively. It is essential to accurately forecast the battery health of RSUs to assess their dependability and running time. The primary duty of the BMS is to estimate the status of the battery and its dynamic operating limits. However, achieving an accurate and robust battery state of charge remains a significant challenge. Referring to that can help road managers make alternative decisions, such as replacing the battery before the RSU power source gets drained. The proposed method can be deployed in other remote WSN and IoT-based applications.

## 1. Introduction

Wireless sensor networks (WSN) and communication technologies are emerging applications that allow real-time monitoring of different communication nodes. Today, complex systems and multiple interacting devices in the intelligent transport system (ITS) generate massive amounts of data. The IoT can ease the implementation of traffic management systems (TMS). The combination of ML and the IoT has stimulated the interest of many researchers since it can process enormous amounts of data suitable for solving complex real-world traffic control problems such as traffic congestion at road intersections [1].

Congestion in urban areas characterised by increasing traffic rates is a major concern for transportation management. One of the most important goals of global transportation research is to optimize traffic flow by using travel time, vehicle density information, information gathered by vehicles and the RSUs [2]. With the introduction of loop detectors, radar, cameras and other sensors, road traffic data can be sent to the traffic control center. RSUs can collect traffic flow data on road segments of intersections and send this information to concerned bodies for further data usage [3]. A smart construction architecture that makes use of IoT to control the performance of all technological systems was proposed in order to achieve energy efficiency [4].

The Government of Rwanda (GoR) has realised the potential of emerging technologies and started using innovative technologies in road transport. Road camera units are being imposed on roads to capture vehicles exceeding the maximum allowed speed limits on a particular road segment. Moreover, different roadside nodes are being deployed for other uses such as traffic light scheduling at junctions, monitoring both traffic and greenhouse gas produced by vehicles [5,6]. For these parameters to be monitored, RSUs require sufficient power and the location at which the RSU is fixed requires a good signal and fewer signal barriers such as buildings. Experiments have been conducted to determine the best communication technology that can be used for indoor localisation systems and applications.

Several works have focused on network topology and developing algorithms to reduce power consumption in WSN while improving network lifespan. The power consumption and RSSI, as they are some of the characteristics of indoor localisation, were examined [7]. However, Wi-Fi was found to use higher power than BLE, Zigbee and LoRaWAN, meaning that it is not preferable for use in a system that relies on batteries to function. As electricity is one of the big challenges not only in the least developed countries, continuous monitoring of those RSU nodes relying on the battery power by predicting their batteries’ lifespan is needed to avoid the failures that might be raised from deploying these units and failure to obtain the data that were to be generated from those road units. This research aims:

- To develop a GSM/GPRS-based RSU that communicates the data to the database while powered by the battery;
- Evaluate the effect of RSSI and the data transmission frequency on the current consumption of a battery-based RSU; and
- Identify the appropriate ML method to forecast the battery discharging time of the RSU in order to replace the battery.

The GSM signal strength in urban and rural locations is not always the same, and the RSSI might also vary by location in urban or rural areas. This work shows how poor signal and frequency of data transmission contribute to the reduction of the expected battery running time of a GSM-based RSU. The RSU data communication begins to degrade as the battery power becomes low. The major contributions of this paper are the following:

- To propose an IoT-based RSU’s data communication architecture;
- To determine the feature importance of RSSI and data communication frequency on the battery discharging time of GPRS-based RSUs; and
- To identify the best ML model for the prediction of the RSU’s battery running time.

The results of this study will support the design and development of expected battery running time prediction using features of IoT and ML. This system could be used by road authorities and decision makers for the adaptation to increasing RSUs’ quality of service. The remaining sections of this paper are as follows: Section 2 discusses the related literature, Section 3 presents materials used during our experimentation and the methods to achieve the results. Section 4 discusses the experiment and its results. Finally, in Section 5, the conclusions with the future work directions are presented.

## 2. Related Works

Vehicle-to-Everything (V2X) communication and its constituents Vehicle-to-Vehicle (V2V) and V2I communication are emerging technologies for addressing the pressing issues of road transport management. These communication technologies are being used in transport for different purposes including collision avoidance, traffic signal control at the intersection, reducing congestion and many more [8]. All these technologies are associated with wireless technologies such as long-range (LoRa), Bluetooth Low Energy (BLE), Zigbee and Wi-Fi communication that consume power [9,10]. These wireless technologies are accused of having some disadvantages in their uses such as that Zigbee can introduce higher latency and LoRa has multi-hop communications and high costs [11]. An advanced objective function zero (AOF0) routing algorithm together with a datagram transport layer security protocol in a fog network were proposed and revealed to have good performance, promising for it to be successful in power and delay reduction. The proposed AOF0 not only extends the life of the network but also includes a simple method for lowering network overhead compared to other objective functions [12].

Technologies for V2X communication face various challenges during data transmission including power consumption and latency resulting in poor transmission throughput [13,14]. The V2I communication system was created to ensure the reliable and autonomous operation of the systems. RSUs are at the heart of vehicle monitoring by allowing information sharing through sending the alert message to other vehicles around and communication with the traffic control center [15]. IoT, WSN, LoRa technology and cellular wireless networks have enabled vehicles and RSUs to collect real-time data for analysing the driving behavior of the drivers [16]. Within a few years, it is anticipated that there will be numerous vehicles on the road with Wi-Fi [17].

Various methods are currently being adopted to power the roadside nodes. RSUs are substantial hardware and have a considerable effect on the infrastructure of the vehicular ad hoc networks (VANET). The RSUs’ consistent operation ensures the effectiveness of the VANET’s goals. Lithium-ion batteries have become widespread in recent years. RSUs are being powered through lithium-ion batteries, acid batteries, solar and energy harvesting-based methods to communicate with road vehicles [18]. They are the most dominating in the market as they are economical for use in various products [19]. However, for effective data sharing, RSUs need to be powered enough. RSU sleep scheduling and joint placement in a VANET environment were proposed to optimize RSU power consumption [20]. Energy consumption optimisation with a green scheduler consisting of prediction, ON/OFF and evaluation algorithms was suggested to lower the RSU’s power consumption [21]. A remaining-useful-life prediction method was proposed to forecast the expected battery lifespan using a gathered recurrent unit neural network and soft-sensing method while reducing failure risks [22].

Battery capacity estimation is one of the noteworthy features of the BMS. Battery ability indicates the battery’s maximum storage capability, which is critical for the battery’s state of charge (SOC). A hybrid method consisting of a convolutional neural network and attention mechanism was proposed to predict the SOC of lithium-ion batteries [23]. An advanced battery management system based on artificial intelligence was proposed; the method uses advanced power electronics and AI to recognize early signs of battery degradation [24]. There have been efforts to predict the health of a battery. Lithium-ion batteries are used as the main power sources in electric vehicles, mobile phones and uninterruptible power supply devices as they have high-energy, high-power density and a long cycle life [25].

The performance of the LSTM algorithm for predicting the state of health of rechargeable lithium-ion batteries was compared to that of an RNN-based algorithm [26]. For the efficient operation of a BMS applied to a lithium-based UPS device, a deep learning-based model that can forecast battery life was investigated [27]. The relationship between RSSI and the battery lifespan in WSN was evaluated by using mTOSSIM, which permits an estimation of the power consumption in a long-term analysis as well as the battery lifetime of the devices [28]. The reinforcement learning technique was used to evaluate the radio environment while designating appropriate ways to reduce overall power consumption and increase reliability resulting in battery lifetime extension [29].

In [30], the authors described the conditions of the distribution of WSN nodes. A network was created to increase life expectancy by changing network topology. The method to calculate network life expectancy was analysed and assessed. It was observed that the overall lifecycle of each network can be changed by the developed structure of a network. Both humidity, temperature and other environmental factors have been demonstrated in other studies to have a substantial impact on RSSI in the outdoor environment. The RSSI metric is sensitive to numerous variables that cause it to fluctuate [31]. Studies that worked on V2X revealed that RSSI of any received data shows the quality of wireless links [32]. Moreover, in [33], the authors used Li-ion cells in the experiments to evaluate the impact of both RSSI and the impedance on the power line communication. The impedance and DC power sources output voltage were calculated for the equivalent resulting in the circuit’s output voltage.

In this study, the RSSI and data transmission frequency were considered to evaluate their effects on the RSU battery power consumption. Both RSSI and current consumption by transmission frequency were gathered from the experiment and were used as predictors of RSU’s battery’s expected running time. Electrochemical and thermodynamic reactions to analyse battery ageing were not considered in this work. Moreover, the effects of temperature, humidity and other environmental factors that affect the RSSI value were assumed to be out of the scope of this work. Impressed by the problems mentioned above, this paper aimed to measure an RSU battery’s power consumption, describe the IoT architecture to send the data to the road traffic management center and evaluate the appropriate ML method to forecast the battery running time of the RSU before it dies and needs to be replaced.

The GSM signal strength in urban and rural locations is not always the same; the RSSI might vary by location in urban or rural areas based on various factors. The overall objective of this paper was to determine the RSSI of different locations while also determining the power consumption by RSUs in our targeted locations. This work aimed to show how poor signal and frequency of data transmission contribute to the reduction of the SOC of GSM-based RSUs and identify the correlation between RSSI and current consumption. The RSU data communication begins to degrade as the battery power becomes low. With the introduction of sensors at intersections, it is now possible to implement an adaptive traffic signal control strategy to optimise traffic flows by adjusting signal timing in real time based on traffic conditions.

In the least developed countries, developing a WSN for IoT applications that work on LoRA and other technologies might cost a lot of money. Hence, as in many countries, the GSM cellular network is well established, building applications and systems that are cellular communication-based might cost less as the network infrastructures have already been established. The East African Community (EAC) is adequately covered by the mobile network. According to the ITU, GSMA mobile connectivity is estimated at 76%. One-area network membership by Rwanda, Kenya, South Sudan and Uganda has made communication among EAC Partner states simple. The ageing phenomena and their stimulates must be understood and communicated in order to predict battery health. The low availability of electricity services hinders the EAC region due to the excessive cost of supply, insufficient interconnections and relatively high distribution losses of electricity.

## 3. Materials and Methods

### 3.1. Study Area

The City of Kigali (CoK) is the most active and progressive among other cities in Rwanda. The city is located at a latitude of 10°58′ S and a longitude of 30°07′ E. The CoK has a total area of 730 km${}^{2}$, a population of 1.2 million and a plus 4% urbanisation annual growth rate. In recent years, the GoR has undertaken the preparation of several urban development plans in the sectors of planning, transport and infrastructure. Currently, the CoK counts 20 junctions that are signalised. However, they are inefficient as the signals are not dynamically linked to traffic sensors to respond to changing demand. Moreover, these junctions are having traffic cameras as RSUs that are mainly used to capture drivers that violate road traffic rules. Experiments were conducted at five signalised junctions to evaluate the impact of RSSI and the frequency rate at which the data are transmitted to the database on the current consumption of battery-based RSUs considered to be GPRS-based sensor nodes.

**Figure 1:**
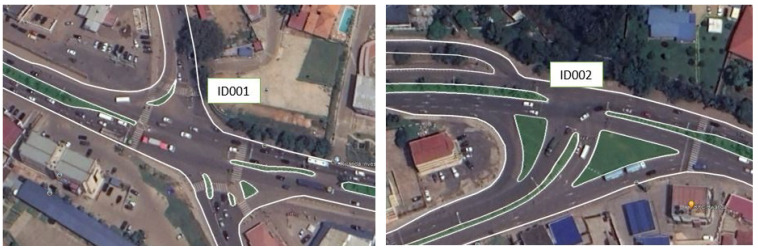
Two consecutive junctions at Remera.

**Figure 2:**
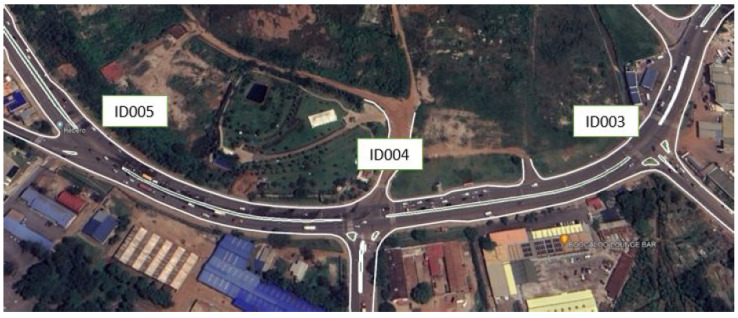
Three consecutive junctions at Rwandex.

The junctions were chosen based on the fact that they are the busiest intersections in the CoK. One of the common characteristics of these junctions is that they are adjacent. The intersections were given identification numbers (ID), ID 001 (Lat. 1°57′39.13″ S, Long. 30°07′12.91″ E), ID 002 (Lat. 1°57′34.18″ S, Long. 30°7′0.88″ E), ID 003 (Lat. 1°58′7.73″ S, Long. 30°5′17.93″ E), ID 004 (Lat. 1°58′9.99″ S, Long. 30°5′9.62″ E) and ID 005 (Lat. 1°58′7.77″ S, Long. 30°5′1.52″ E), respectively. The entire average delay time at these junctions would be reduced only when coordinated control of traffic signals is applied and that has to depend on the traffic situation captured by the RSU imposed on the junctions. As the traffic flow rate depends on the period of the day and the season, respecting real-time traffic flow-based traffic signal scheduling could increase the effectiveness of the battery state of charge [34]. Figure 1 and Figure 2 show the aerial layouts of our areas of study.

### 3.2. Methodology

This part details the methodology adopted for measuring RSSI and battery current consumption based on the data transmission frequency at five junctions where two experiments were conducted. The first was to measure the RSSI. The sampling interval of RSSI was every 20 s at each junction, and the overall data average was considered to be the RSSI at the specific junction. The second experiment was to measure the current consumption of the RSU at each junction with various frequencies of data transmission.

#### 3.2.1. Road Side Unit

**Figure 3:**
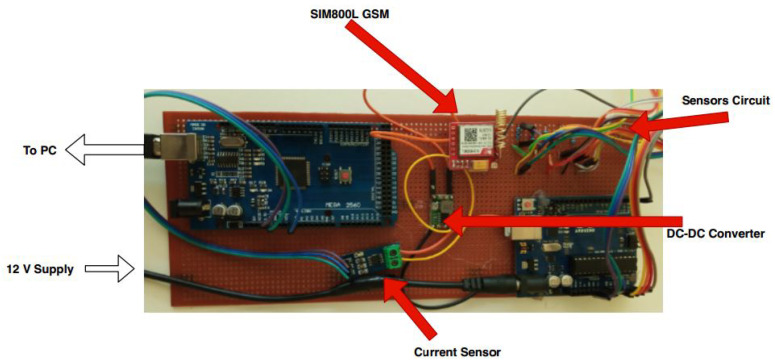
Internal circuity of the RSU.

The RSU experiment setup was composed of the microcontroller, SIM 800L, GSM/GPRS module, GPS module, current sensor, IR sensor and the 12 V battery supplying the RSU node. Figure 3 shows the internal part of the RSU. This is the main part of our experiment as it is the one that senses data and sends them to the remote database for further analysis:

- Microcontroller: This is the core part of data acquisition. The actual programmable board serves as the central nervous system, and flow chart logic is built here. The GSM/GPRS module is linked to the microcontroller, allowing for the connection of sensing devices to the board. The GSM/GPRS module transmits data as well as other information to the database;
- GSM/GPRS Module: To transmit the data to a remote web server, an Arduino Uno module interface was used. This GSM/GPRS module works with GSM frequencies in the range of 850–1900 MHz. The module’s protocol allows data to be sent to the database via the GSM network;
- GPS module: The GPS module interoperable with the Arduino Uno was using satellite data to locate the source of data. These data are of invaluable usage by road managers and transport in charge to quantify traffic flow based on RSU battery power consumption;
- IR Sensor: An infrared sensor can be used to detect motion or objects, based on the perceived parameter in the environment. The infrared sensor module contains both the transmitter and receiver. An IR LED serves as the transmitter, while a photodiode serves as the receiver [35]. A system that uses IR sensors to measure traffic density and a microcontroller to manage the switching of traffic lights in response was proposed [36]. This has shown that the dynamic traffic-controlling system outperformed the static and conventional traffic-controlling systems;
- Current sensor: This sensor measures current ranging between −5 A and 5 A. A model-based sensor error detection and isolation for Li-ion cell batteries were used to determine battery degradation. An online load current and state-of-charge estimation and the findings offered valuable knowledge for reducing the structural complexity and expense of using lithium-ion batteries in the future [37].
- Lithium-ion battery: A 12 V/7 Ah was used to supply current the node.

#### 3.2.2. Received Signal Strength Measuring

The RSSI determines the strength of the signal power received in a certain place. The RSSI quality is mainly affected by the distance between the transmitting and receiving devices. However, other factors also affect the RSSI value, including the positioning, sensor geometric orientation, climatic influences and so forth that were out of this work’s scope. The power, responsiveness and orientation of the antenna with respect to the transmitter significantly affect the received signal of the receiving node [38]:

(1) RSSI=−10nlog(d)+V

where *d* presents the distance between the transmitter and receiver’s antennas, *n* is the path-loss constant that varies based on the environment, and *V* is the fixed constant accounting for system losses.

To gain the RSSI values at the junctions in Figure 1 and Figure 2, the GSM module was connected to the microcontroller. Python codes were written in the microcontroller where the path on the computer to store the data that responded to the GSM module from the base station was created, and the data were saved in the comma-separated values (CSV) file to the specified path. The AT+CSQ AT command was sent to the GSM module connected to an Arduino, and the GSM module was responding to the signal quality in the timing advance (TA) value through the nearby base station of the GSM network. Table 1 describes key AT commands used to gain the RSSI values at the junctions.

#### 3.2.3. Current Consumption Measuring

**Figure 4:**
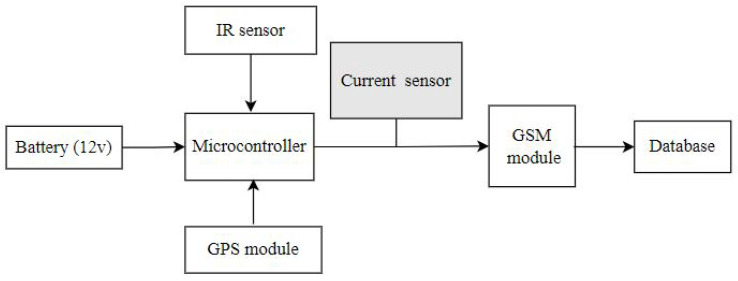
Current acquisition process.

Degradation affects the performance of the battery [39]. The current sensor was used to acquire the current consumed by the RSU components. In a general way, the power consumption of the RSU was looked at in three ways: the power consumed by the sensing units (each component/sensor of the RSU), the power consumed by receiving units while processing the data and the power for radio unit (GSM) when it is transmitting the processed and packed data to the database; this RSU power consumption is calculated in Equation (2). The current consumed by the node’s components while the GSM module was OFF was measured and recorded. Thereafter, the GSM 800L module was connected to the circuity in order to connect to the GSM network for data communication, and the variation in current consumption was observed. The variation caused by the radio unit module was considered, as the hypothesis was that the battery running time depends on both the RSSI and the frequency at which the data are transmitted to the database, assuming that the current consumed by the sensing part does not change:

(2) $$C_{Tot}=C_{Sens}+C_{Proc}+C_{Trans}$$

where *C*${}_{Tot}$ is the total current consumption of the roadside unit, *C*${}_{Sens}$ is the current consumption of the sensing unit, *C*${}_{Proc}$ is the current consumed by the processing unit and *C*${}_{Trans}$ the current consumed by the radio unit (GSM module). Figure 4 details the circuitry current consumption by GSM/GPRS when transmitting the data to the database.

The RSU current consumption (*C*${}_{Tot}$) increased or decreased based on the increase or decrease of the processing unit’s current consumption (*C*${}_{Proc}$) and the current consumed by the radio unit (*C*${}_{Trans}$). The *C*${}_{Trans}$ varies based on the change in data transmission frequency. The *C*${}_{Trans}$ depended on the RSSI value of the location where the RSUs were located. The current consumed by the sensing unit (*C*${}_{Sens}$) remained constant as the RSU components were the same at different locations. In our experiment, the current data were captured and then transmitted to the database every 20 s, 40 s and 60 s, respectively, where, later on, the data were extracted in CSV format and analysed in Python to evaluate the best ML model for the prediction.

Different researchers used current sensors to determine battery degradation by developing model-based sensor error detection and isolation for Li-ion cell batteries. An online load current and state-of-charge estimation were proposed and their findings offered valuable knowledge for reducing the structural complexity and expense of using lithium-ion batteries in the future [37]. Batteries’ capacity can be measured in the amount of current consumed and capacitance decline trend. When the capacitance of the battery drops below 80% of its nominal capacitance, the endpoint of the battery’s life is reached [40,41]. At this time, the battery’s failure threshold is set to 80%. Therefore, in this work, the test bed was powered by a 12 V battery (12 V/7 Ah), and the state of health (*SOH*) is determined by Equation (3):

(3) $$SOH=\frac{C_{t}}{C_{m}}$$

where *C*${}_{t}$ denotes the capacitance of tth cycle, and *C*${}_{m}$ represents the nominal capacitance of the battery. Considered deterioration of battery performance was reflected by the capacitance of batteries. While the definition of the remaining useful life (*RUL*) of the battery here was considered for our 12 V battery, the work time before the battery drops to the failure threshold was given by Equation (4):

(4) $$RUL=C_{m}*0.8=9.6Ah$$

Hence, the prediction of the remaining usefulness of the lithium-ion battery based on previous current consumption data can be calculated by Equation (5), and the predicted *SOH* to reach the end-of-life of the battery here was referred to as a capacity below 9.6 V:

(5) $$SOH_{t+1}^{p}=f\left(\left[SOH_{t}^{r},SOH_{t-1}^{r},\ldots SOH_{t-w+1}^{r}\right]\right)$$

*SOH*${}_{t+1}^{p}$ is the prediction value of step *t*, *SOH*${}_{t}^{r}$ is the observation value of step *t*, and *w* is the length of the slide window.

#### 3.2.4. RSU Data Communication

**Figure 5:**
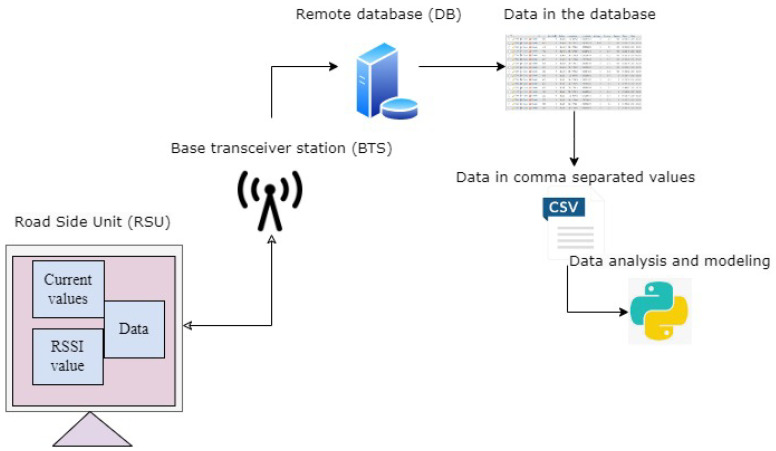
System architectural diagram.

The layers of the architecture in our proposed work are the data acquisition layer (RSU), the network connectivity (BTS) that was built on the GSM communication and the service layer for the traffic manager and other concerned users to visualise and export the data. The RSU is capable of transmitting the data to the remote database. The roadside unit collects the data, packs the data and sends them to a remote database where the data can be extracted or exported in any format for further analysis. Different sensors can be integrated into the RSU. The current measured and sent to the database was read when the RSU processing part started to send the data to the database using GPRS protocols. Figure 5 presents the architectural diagram of the data communication.

Table 2 describes the acquired data, where “ID” represents the intersection identification number, and “Longitude” and “Latitude” are the geographic locations of the intersections, “RSSI” is the average value of the signal at the area and the average current consumed within that area by the interval of data transmission to the database. The current consumption is estimated in amperes, and it is symbolised by A.

**Figure 6:**
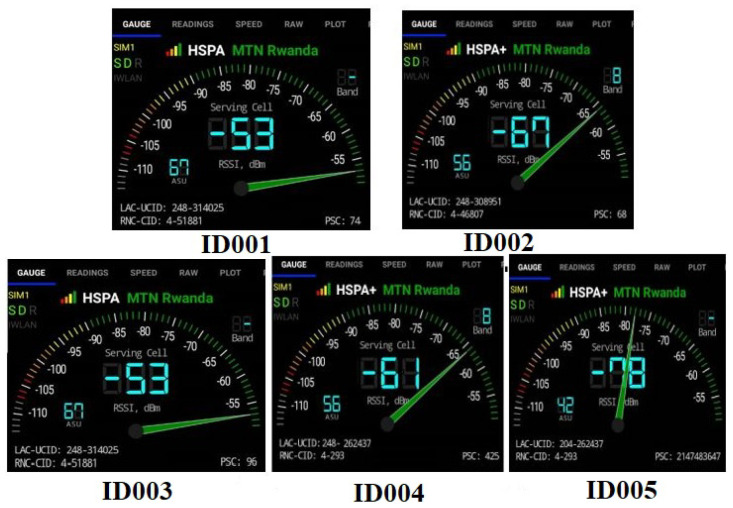
Various RSSI by location and operator ID.

**Figure 7:**
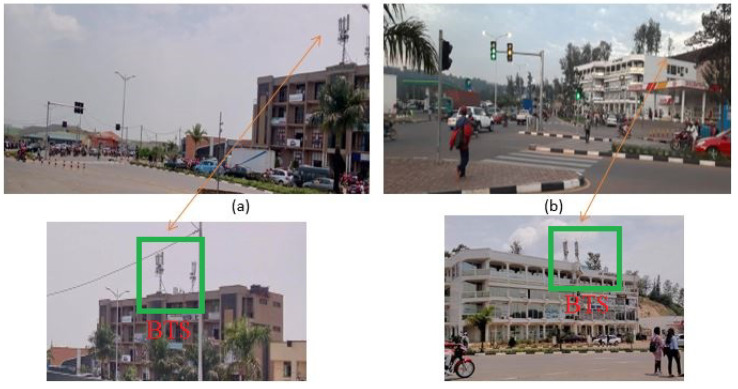
Position of BTS at the junctions ID001 and ID003. (**a**) the BTS positioned at 50 m from intersection ID001 and (**b**) the BTS positioned at 30 m from intersection ID003.

Figure 6 shows the readings by the network cell info Android application that was used to identify the BTS location. The application was used to determine network signal strength and local serving cell information. The network operator and operator ID information at each location were collected and shared with the network operator to help us in identifying the geolocation of the BTSs. This information was used later on to measure the distance from RSU to BTS. The distances from ID001 to BTS were 50 m, ID002 to BTS (30 m), ID003 to BTS (30 m), ID004 to BTS (40 m) and ID005 to BTS (50 m), respectively. Figure 7 shows where the BTS were located on some of the junctions in the study.

### 3.3. Data Preparation for Forecasting

The two models were evaluated by using training and validation datasets to predict the battery health of the RSU node based on the RSSI value of where the RSU is imposed and the current consumed when transmitting the data to the database. The dataset was divided into training and testing datasets. The training dataset comprises 70% (11,880) and the testing dataset consisted of 30% of the total recorded data (16,973) during our experiments. Four categories, namely RSSI value, and current consumption when data are transmitted every 20 s, 40 s and 60 s, respectively, were considered as predictors. They were labelled as RSSI (dBm), current_20 s (current values when data were being sent every 20 s), current_40 s (current values when data were being sent every 40 s) and current_60 s (current values when data were being sent every 60 s), respectively. The data values of these attributes were stored in the database and were later on exported in the form of CSV files to apply the ML models to the data.

### 3.4. Machine Learning Models

In this work, two prediction methods were used to evaluate the best prediction model for battery SOC using both predictors’ RSSI values and the frequency of the data transmission to the database. The SVM and RF for classification and prediction were used as they are technologies that can reduce road authorities’ time loss by offering them RSU’s battery charge insights. ML methods for predicting battery health are indeed a kind of data analysis that automates the creation of analytical models. It is based on the idea that systems can learn from data, discover patterns and make judgments or forecasts with minimal or no human intervention. The first step was to collect data. Measurable parameters such as RSSI, the frequency at which data were communicated to the central database and current data were recorded during RSU operation. The next stage was to extract the characteristics of the battery to be discharged. The third was to train an ML model to describe the link between the extracted variables and battery running time.

Feature extraction is a vital process that has a substantial impact on battery performance. More meaningful and precise input data will result in more relevant and precise predictions. To train the ML algorithm, some researchers used battery health model metrics such as electrical resistivity, series resistance and polarisation capacitance as input features. This method necessitates the use of an electrical model using online state estimation techniques [42,43]. During data preparation, the following steps were followed: data collection, pre-processing, model training and selection, model training and selection, model feature selection and then the prediction.

- Data collection: At this stage, the RSSI and current consumed values from various locations (RSUs) were gathered. The data provided insights on the variation of RSSI by location, and the current consumed when data are transmitted with respect to different frequencies of the data transmission to the database;
- Data pre-processing: Data were pre-processed to remove missing values and irrelevant values. After this, features to predict RSU’s battery SOC were carried out, features were extracted and data were split;
- Model training: The appropriate ML algorithms based on the current consumption problem were evaluated. SVM and RF have been considered. This was followed by training both models on the pre-processed data;
- Model evaluation: The performance of each trained model was evaluated using metrics such as mean absolute error (MAE) and the $R^{2}$;
- Feature selection: This consisted of selecting the most relevant features that strongly influence the current consumption;
- Prediction: The models were applied to the collected data to make predictions about current consumption and behaviours based on changes in the transmission frequency of the data to the remote database.

**Figure 8:**
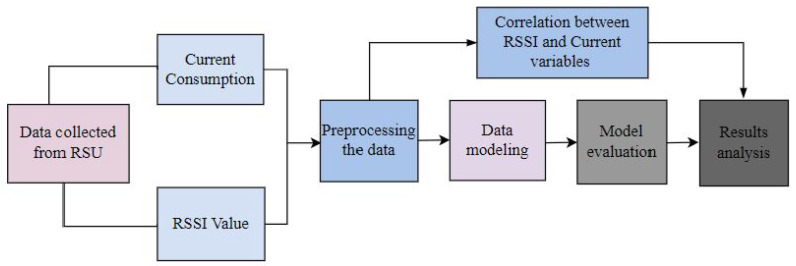
Data collection and processing.

Figure 8 shows activities in the process where RSSI and current data collected were used for model evaluation. The method for predicting the RSU’s battery health was depicted using ML models.

#### 3.4.1. Support Vector Machine

SVM, which is a supervised ML algorithm, was introduced by Vapnik in 1995 to solve classification and regression problems. The SVM is one of the supervised learning models that investigate data and identifies data samples used for classification. Assuming that battery power consumption is a regression problem *X*, RSSI ($x_{1}$), the frequency at which the data are sent to the TMC are the time stamp 1 ($x_{2}$), time stamp 2 ($x_{3}$) and time stamp 3 ($x_{4}$) are considered as input (predictors) for SVM, in order to understand how the response *X* depends simultaneously on the predictors in our context assumed to be ($x_{1}$), ($x_{2}$), ($x_{3}$) and ($x_{4}$). Given the training data consisting of the input matrix *X* = [*x*${}_{1}$, *x*${}_{2}$,…, $x_{n}$] and an output vector *Y* = [*y*${}_{1}$, *y*${}_{2}$,…, $y_{n}$], the SVM construct an optimised linear regression through mapping the input vector *x*. SVM in ML approaches includes a set of learning methods and shows better results [44]. SVM performs linear and nonlinear classification. SVM supports linear and nonlinear regression applications [45].

#### 3.4.2. Random Forest

RF is a prediction method categorised as ensemble learning that integrates multiple decision trees. It is one of the data mining tools used in the ML framework. It can be used for both classifications, regression problems and time series forecasting. The RF classifier is a collection of decision trees derived from the random training set. It combines the votes from various decision trees to figure out the final class of the test object. It has a series of decision paths from the node to the last leaf safeguarded by a sub-feature. Prediction is a sum of individual features and the mean value of the topmost region covered by the training set. Given a training dataset A = (x${}_{i}$, y${}_{i}$), *i* = 1, 2,…, n, (X,Y)$\in R^{m}\times R$, the input matrix *X* consists of *n* samples with *m* features, and the output *Y* is a target vector. The RF adopts the bootstrap re-sampling method to form *N* tree sample sets (S${}_{k}$, *k* = 1, 2,…, N tree) randomly from the original sample set *S*, the number of elements of S${}_{k}$ is the same as that of *S* (where k is the current number of iterations). In bootstrap samples, approximately one-third of the data in the original sample set *S* which are called out-of-bag (OOB) data are not drawn, and the remaining data are called in-bag data [46]. Random survival forest (RSF) being an extension of RF to regular random forests, independent bootstrap sampling was used to handle multicollinearity while examining lifetime prediction of lithium-ion batteries

### 3.5. Models’ Validation

Various metrics for evaluating regression models have been developed and used. The performances of the models were assessed by using the usual model performance metrics mean squared error (MSE), mean absolute error (MAE), root mean squared error (RMSE) and the $R^{2}$ [47]. The MAE, MSE, RMSE, and the $R^{2}$ were the indicators used to determine the performance of the SVM and RF models [48]. The two key parameters that determine the RF model’s capacity to estimate are the number of trees created and the variables used to construct each tree. The model’s mean square error calculation is calculated by the *OOB*, and this is the method for measuring the prediction error and computing the variable importance [49]. Equation (6) is used to calculate the error:

(6) $${\mathrm{MSE}}_{OOB}=\frac{1}{n}\sum_{i=1}^{n}{\left(O_{i}-P_{iOOB}\right)}^{2}$$

The MSE is the average of a set of errors, and it is given by the Equation (7):

(7) $$\mathrm{MSE}=\frac{1}{n}\sum_{i=1}^{n}{\left(y_{i}-{\hat{y}}_{i}\right)}^{2}$$

where *n* is the observation number, and $P_{iOOB}$ is the average of the *OOB*’s predictions across all the trees. In this paper, the training dataset comprises approximately 70%, and the testing dataset consists of 30% of the total recorded data. The MAE given by the Equation (8) is a risk metric corresponding to the expected value of the absolute error:

(8) $$\mathrm{MAE}=\frac{1}{n}\sum_{i=1}^{n}\left|y_{i}-{\hat{y}}_{i}\right|$$

The RMSE, which is the standard deviation of the residuals (prediction errors), is defined by Equation (9):

(9) $$\mathrm{RMSE}=\sqrt{\frac{\sum_{1}^{n}\left(y_{i}-{\hat{y}}_{i}\right)}{n}}$$

The $R^{2}$ values range from zero to one [0, 1]. Zero (0) illustrates that the current consumption can not be predicted based on the historically recorded current, while One (1) implies the perfect prediction and is given by Equation (10):

(10) $$R^{2}=1-\frac{\sum {\left(y_{i}-{\hat{y}}_{i}\right)}^{2}}{\sum {\left(y_{i}-{\bar{y}}_{i}\right)}^{2}}$$

where ${\hat{y}}_{i}$ is the predicted value of the $i^{th}$ sample, and $y_{i}$ is the corresponding true value for the total *n* sample.

## 4. Results and Discussion

After generating the data, the Jupyter Notebook was used for data analysis and graphical data presentations. In the following section, we present all steps conducted during our experiments where Arduino code is used for transmitting current data to a remote server in different conditions of how often data are transmitted. Some assumptions were made: (1) all RSUs at the junctions are fixed so that there are no changes in RSSI, (2) the RSU transmit the data in a given interval of time to reduce power consumption, and (3) the IR sensors are used to capture the traffic flow data.

### 4.1. Received Signal Strength Indicator

In five locations where the experiment was carried out, it was realised that the RSSI varies from location to location. In our experiment, the receiver is a GSM module (SIM 800L); to obtain information about its RSSI at a particular location, we were sending an AT command (AT+CSQ=?) to it with the help of an Arduino board, and the module was responding with its current RSSI. However, there was no high variation between the RSSI values in the areas of interest. The signal quality was excellent at four locations ID001, ID002, ID003 and ID004, respectively, and good at one location ID005. At the two locations (ID001and ID003), the RSSI was similar, leading to no significant difference in the power consumption at both locations. Having assumed that battery power consumption is a regression problem, the RSSI and frequency at which the data are sent to the database were considered predictors for both models.

### 4.2. RSSI versus Current Consumption

**Figure 9:**
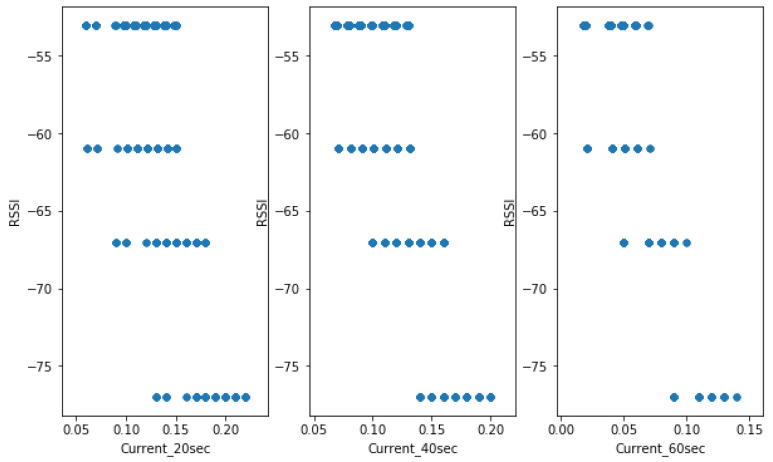
Current consumption vs. frequency of data transmission.

In our experiment, it was observed that, when RSUs were sending data after every 20 s, the module was consuming a high amount of current compared to 40 s and 60 s. The consumption was also varying based on the RSSI value of the locations. Figure 9 shows the correlation of the variables. The current consumption will increase if the RSU is fixed at a location characterised by poor signal quality. To minimize current consumption, the RSU will not have to send the data often. The worse the RSSI, the higher the power is consumed. As the data were transmitted at a high frequency, the power consumed increased. As the RSSI tends to marginal condition (−109 dBm), the data transmission frequency has to be minimised in another RSU node to consume less current. On the contrary, the more excellent the RSSI (−53 dBm), the less the RSU will consume the power. From the figure, when the RSSI is not good (−77 dBm) and the data are sent first (every 20 s), the current consumed is high between 0.13 and 0.23 A compared to when data are sent every 40 s (0.14–0.20 A), 40 s (0.08–0.14 A), respectively, meaning that, when the RSSI is not good, the option to reduce power consumption is by increasing the interval time to transmit the data to the database.

#### 4.2.1. Collinearity of the Data

**Figure 10:**
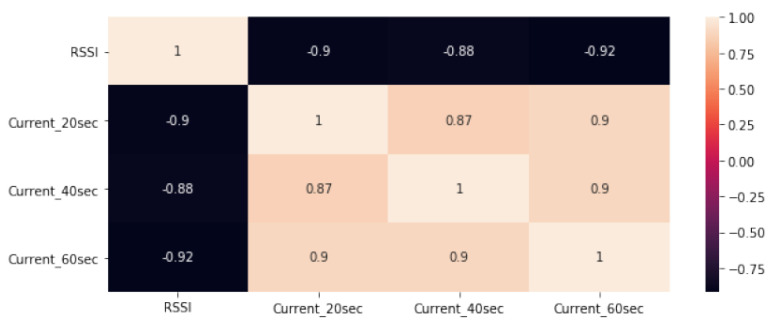
Pearson correlation between RSSI and current variation.

The collinearity was computed to assess the correlation between the RSSI, current_20 s, current_40 s and current_60 s variables. Hence, to predict the working time of the battery, it is necessary to apply a direct relationship by combining the variables that might affect battery health. Figure 10 shows the correlation of the variables. It also shows how all parameters are related to estimating the average expected battery running time.

#### 4.2.2. Data Pairwise

**Figure 11:**
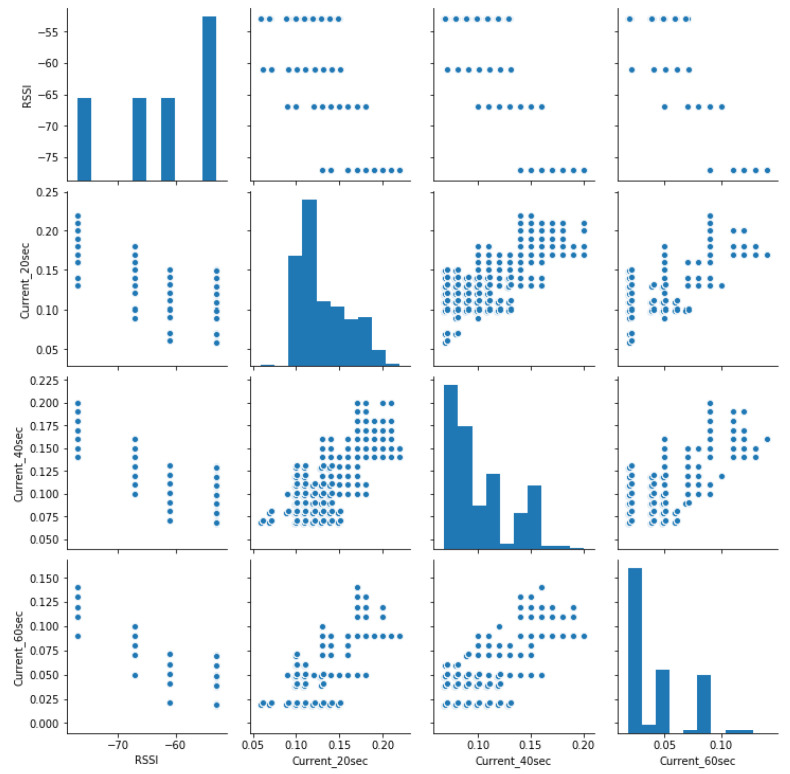
Pair plot of the used variables.

Figure 11 shows the pair’s plot that visualises the current distribution. Despite the fact that current consumption is the challenge, the lower RSSI, the higher the chance for the RSU battery to reach its discharging time. The higher transmission frequency also reduces battery running time. Both parameters affect the system’s functionality, according to our observations during our experiments and analysis.

#### 4.2.3. Case 1: Data Are Transmitted Every 20 s

**Figure 12:**
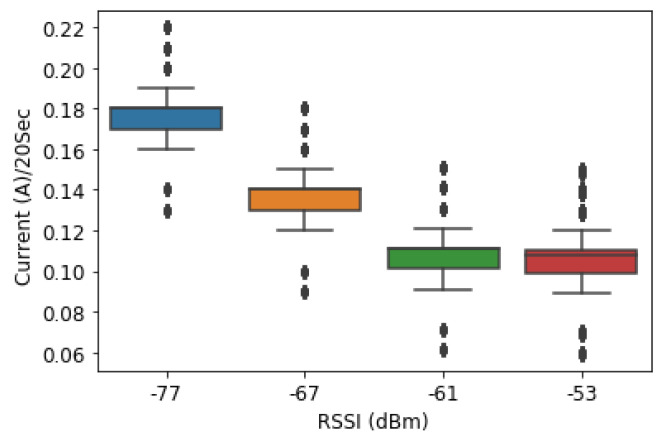
Current consumption vs. RSSI in 20-s time stamps.

In Arduino coding, the minimum allowed time delay given for the module to receive and respond to AT commands for GPRS varies from 20 s. This is the reason we started at a minimum of 20 s; then, we wrote GPRS code for transmitting data to a remote database every 20 s. Figure 12 shows that the current consumed by the RSU increased as the frequency of data transmission was at a higher frequency (20 s). As in the figure, the lower the RSSI values (−77 dBm), the higher the current consumption was (0.17 A).

At the location where the RSSI was −77 dBm, the current consumption when the RSUs transmit the data every 20 s was 0.17 A on average compared to −67 dBm (0.13 A), −61 dBm (0.11 A) and − 53 dBm (0.10 A and 0.09 A), respectively. The current consumed by the GSM module when connecting to the GSM network to transmit the data every 20 s was higher at ID005 (−77 dBm) compared to ID001 and ID003 where the RSSI was −53 dBm.

#### 4.2.4. Case 2: Data Are Transmitted Every 40 s

In the same way as the previous experiment in the case of the 20 s rate, we wrote some GPRS code for transmitting data to a remote database every 40 s. The average amount of current consumed for SIM800L was varying based on the RSSI of the location and was better compared to the previous case 1. For the location where the RSSI was −77 dBm, the current consumption when the RSUs transmit the data every 40 s was 0.15A on average compared to −67 dBm (0.11 A), −61 dBm (0.08 A) and −53 dBm (0.10 A and 0.08 A), respectively.

**Figure 13:**
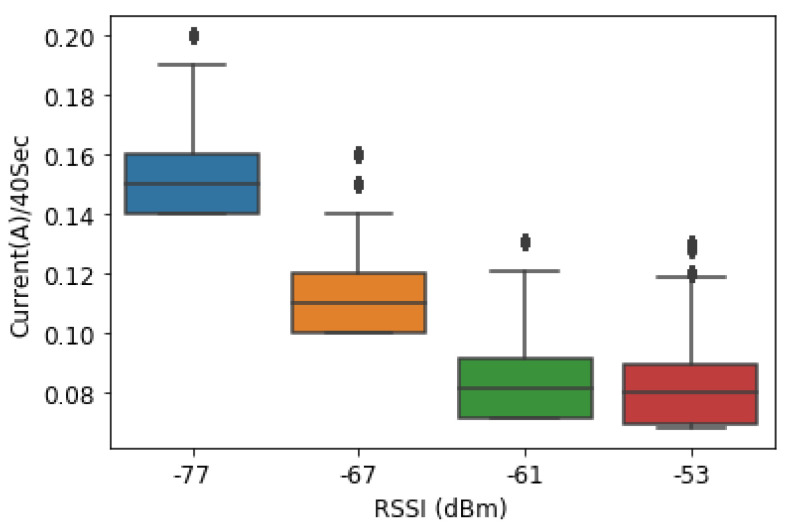
Current consumption vs. RSSI in 40-second time stamps.

Comparing Figure 12 and Figure 13, at the same location that was ID005 (−77 dBm), when the data were transmitted to the database, the GSM in 20 s, the current consumed was 0.17 A compared to 0.15 A when it was every 40 s.

#### 4.2.5. Case 3: Data Are Transmitted Every 60 s

**Figure 14:**
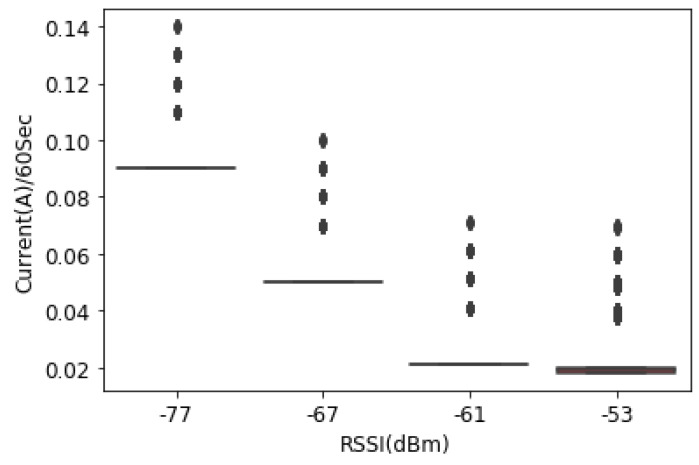
Current consumption vs. RSSI in 60-second time stamps.

GPRS code for transmitting data to a remote database every 60 s was written. According to Figure 14, the average amount of current consumed by SIM800L was significantly reduced. At the location where the RSSI was −77 dBm, the current consumption when the RSUs transmit the data every 60 s was 0.04 A on average compared to −67 dBm (0.04 A), −61 dBm (0.03 A) and −53 dBm (0.01 A and 0.01 A), respectively. This leads to the fact that, when the transmission frequency is reduced, the current consumption reduces at both locations where the RSSI is good and where it is not adequate.

### 4.3. Comparative Analysis of the Models

**Figure 15:**
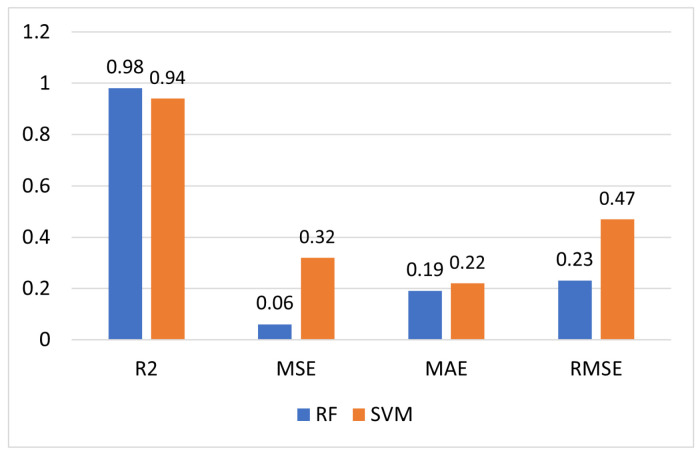
Comparison of the performance metrics.

To validate the prediction capability of the suggested models, the accuracy of the RF versus SVM model was assessed to evaluate their performance. RSSI values, current consumption and data transmission frequency were the inputs to the models. $R^{2}$ is the measure of how the values fit together in relation to the starting values; this is an indication of goodness of how well unseen samples are likely to be predicted by the model. The MAE represents the original-to-predicted value difference, and this is the average absolute difference across our whole data set. The RMSE stands for residual mean square error or prediction error. Compared to the SVM model, RF revealed a higher $R^{2}$ and lower MSE, MAE and RMSE. Figure 15 shows the prediction results from the comparison of the RF and SVM model.

The RF-based prediction model showed an $R^{2}$ = 0.98, MSE = 0.06, MAE = 0.19 and RMSE = 0.23, compared to SVM, which has an $R^{2}$ = 0.94, MSE = 0.32, MAE = 0.22 and RMSE = 0.47. The RF values were preferable to those of SVM, particularly when considering the $R^{2}$ of 98% for the prediction of the current consumption of RSU that communicate based on GSM/GPRS. RF is a pattern recognition method that uses a synchronous learning strategy to build a large number of classifiers and aggregate their results to form the final prediction. RF is capable of performing classification, regression and unsupervised learning.

## 5. Conclusions

In this paper, a battery-based RSU sensor node that communicated the road data using GSM/GPRS protocols was developed. The major objective for the RSU development was to evaluate experimentally and qualitatively the effect of the RSSI and data transmission frequency on the current consumption of the RSU powered by the battery. We have seen that the GPRS protocol consumes a slightly high amount of battery power. Rwanda is commonly known as a 1000 hills country, and the GSM connectivity is covering the whole country; hence, V2I, V2X and IoV applications can easily use GPRS instead of using Lora, which might require other infrastructures. We have demonstrated that the power consumption of the used module depends on how frequently the RSU will be transmitting the data. Hence, in some areas that are not having high traffic flow rates, commonly remote areas, it requires a low frequency of data transmission compared to clouded regions that are commonly found in urban areas where a SIM800L-based RSU sensor node can survive for many days running on a battery. The remaining useful life of the battery was considered for our 12 V lithium-ion battery powering the RSU node, and the working time before the battery drops to the failure was referred to as capacity below 9.6 V as the threshold was 80% of the battery capacity. When the capacitance of the battery drops below 80% of its nominal capacitance, the endpoint of the battery’s life is reached, and, at this time, the battery needs to be taken for recharge. In this paper, we developed a real-time monitoring system that uses IoT features, data processing and an ML prediction model to help estimate the remaining RSU’s battery running time. RF has been proven to outperform SVM. The proposed model will help road managers monitor the status of the batteries at each location; thus, unexpected data losses caused by the batteries’ state of not powering RSUs to transmit the data can be prevented. Through this work, it was shown that integrating IoT and ML for data processing is adequate for processing and analysing sensor nodes’ data from various locations. In the future, a comparison of GSM/GPRS and other communication modes will be compared. Considering the period of the day and season could also reduce the RSU discharging rate. The effect of time, season and traffic signal cyclicity by comparing intersections in urban and rural locations will be conducted to gain more insights into the current consumption. There is also another way of extending this work by developing TinyML at the RSU node to forecast battery health. Environmental factors including the weather and objects can also be considered to evaluate their effects on the received RSSI.

## Figures and Tables

**Table 1:** Description of AT Commands used.

AT Command	Use
AT+CSQ=?	Signal strength report
AT+CREG?	Registration status and access technology of the serving cell
AT+SAPBR=3,1,	Connecting to GPRS
AT+HTTPINIT	Initialises the HTTP service

**Table 2:** Description of RSSI and current consumption by RSU and location.

Intersection	Latitude	Longitude	RSSI	20 s [A]	40 s [A]	60 s [A]
ID001	1°57′39.13″ S	30°07′12.91″ E	−53	0.10	0.08	0.21
ID002	1°57′34.18″ S	30°7′0.88″ E	−67	0.13	0.11	0.05
ID003	1°58′7.73″ S	30°5′17.93″ E	−53	0.10	0.09	0.02
ID004	1°58′9.99″ S	30°5′9.62″ E	−61	0.11	0.08	0.02
ID005	1°58′7.77″ S	30°5′1.52″ E	−77	0.17	0.15	0.09

## Data Availability

The data used to support the findings of this study are available from the corresponding author upon request.

## Abbreviations

The following abbreviations are used in this manuscript:

ACEIoT	African Center of Excellence in Internet of Things
GSM/GPRS	Global System for Mobile/General Packet Radio Service
ITS	Intelligent Transportation System
IoT	Internet of Things
IoV	Internet of Vehicle
RF	Random Forest
WSN	Wireless Sensor Networks
RSU	Road Side Unit
RSSI	Received Signal Strength Indicator
ML	Machine Learning
TMS	Traffic Management System
VANET	Vehicular Ad hoc Networks
V2I	Vehicle to Infrastructure
SVM	Support Vector Machine
